# Idiopathic membranous nephropathy in older patients: Clinical features and outcomes

**DOI:** 10.1371/journal.pone.0240566

**Published:** 2020-10-09

**Authors:** Ji-Young Choi, Ho Jun Chin, Hajeong Lee, Eun Hui Bae, Tae Ik Chang, Jeong-Hoon Lim, Hee-Yeon Jung, Jang-Hee Cho, Chan-Duck Kim, Yong-Lim Kim, Sun-Hee Park, on the behalf of The Korean GlomeruloNEphritis sTudy Group (KoGNET)

**Affiliations:** 1 Department of Internal Medicine, School of Medicine, Kyungpook National University, Daegu, Republic of Korea; 2 Department of Internal Medicine, Seoul National University College of Medicine and Seoul National University Bundang Hospital, Seongnam, Republic of Korea; 3 Department of Internal Medicine, Seoul National University Hospital, Seoul, Republic of Korea; 4 Department of Internal Medicine, Chonnam National University Hospital, Seoul, Republic of Korea; 5 Department of Internal Medicine, National Health Insurance Service Medical Center, Ilsan Hospital, Goyangshi, Gyeonggi-do, Republic of Korea; Kaohsiung Medical University Hospital, TAIWAN

## Abstract

**Background:**

Various factors can affect renal and patient outcome in idiopathic membranous nephropathy (iMN). We aimed to identify predictors of renal and patient survival in patients with iMN, with a special focus on outcomes among older patients.

**Methods:**

We retrieved data on 1,776 patients (mean age 53.0 ± 14.7 years; 1,075 [60.5%] males) diagnosed with iMN from the Korean GlomeruloNEphritis sTudy (KoGNET), a database compiled from 18 centers in Korea.

**Results:**

The cohort included 428 (24.1%) patients over 65 years old. Compared to younger patients, this group had lower hemoglobin and serum albumin levels, a higher incidence of nephrotic-range proteinuria, and higher prevalences of hypertension and diabetes. At last follow-up, complete or partial remission rates were not significantly different between the older and younger groups. Older age (HR: 0.98, 95%CI: 0.97–0.99), elevated hemoglobin (HR: 0.82, 95%CI: 0.72–0.93), high serum albumin (HR: 0.66, 95%CI: 0.44–0.99), and a high estimated glomerular filtration rate (HR: 0.96, 95%CI: 0.95–0.97) at biopsy were good predictors of renal outcomes. Significant risk factors for patient survival were older age (HR: 1.04, 95%CI: 1.01–1.10) and hypertension at biopsy (HR: 2.76, 95%CI: 1.30–5.90).

**Conclusions:**

Older patients with iMN had favorable renal outcomes, but poor patient survival, compared to younger patients. Prognostic information on outcomes in this study might be helpful for optimizing the management of patients with iMN.

## Introduction

Idiopathic membranous nephropathy (iMN) is a leading cause of nephrotic syndrome and one of the major causes of end-stage renal disease (ESRD) [1–3]. The clinical course of iMN is variable; one third of patients experience spontaneous remission, one third experience persistent proteinuria, and one third progress to ESRD [4–6].

Many factors can affect the clinical course and prognosis of iMN. The baseline level of proteinuria in the nephrotic range is an indicator of reduced kidney function in patients with iMN [7, 8]. Patients with high proteinuria levels at the time of biopsy had worse outcomes, in terms of reduction in the estimated glomerular filtration rate (eGFR) or the progression to ESRD. In contrast, patients with proteinuria in the subnephrotic range had excellent outcomes, and did not require immunosuppression [9, 10]. Based on those findings, the Kidney Disease Improving Global Outcomes (KDIGO) guidelines suggested that the proteinuria level should be considered when deciding between active immunosuppressive therapy or conservative treatment [11].

Strong evidence has supported the notion that complete remission of nephrotic-range proteinuria is an indicator of a favorable long-term prognosis [12, 13]. In a study that validated and quantified proteinuria remission as a surrogate for long-term outcome in MN, persistent remission was associated with a reduced risk of deteriorating renal function, and remission durations as short as 3 months were associated with an improved renal prognosis [14]. In addition to proteinuria levels, age and eGFR have been reported as predictors of outcome in patients with iMN. Although younger and older patients with iMN had comparable renal outcomes in a recent Japanese multicenter study [15], other studies showed that older age was associated with inferior renal survival [8, 16, 17]. A low eGFR at disease presentation was also reported to be associated with poor renal outcomes [16, 18]. In addition, an immune response against the phospholipase A2 receptor (PLA2R), which is expressed on the surface of podocytes, could be detected by the presence of anti-PLA2R antibodies in 70–80% of patients with iMN. High anti-PLA2R levels at diagnosis were found to be a significant risk factor for a decline in kidney function and a lower likelihood of spontaneous remission are predictive factors for long term renal outcomes as well as remission [19, 20].

Recently, the KDIGO group suggested an updated risk stratification for patients with MN that require treatment and the risk factors associated with loss of kidney function [21]. Factors associated with a high risk of loss of kidney function included serum creatinine over 1.5 mg/dL; a reduction in the eGFR by ≥20% over any time period during the preceding 12 months, which could not otherwise be explained; and proteinuria >8 g/day for 6 months or more. They also suggested that high anti-PLA2R levels and changes in anti-PLA2R levels were high-risk factors of progressive renal dysfunction.

Although risk factors that could predict iMN outcome have been reported in previous studies [22, 23], they remain controversial. In addition, few multicenter cohort studies with large-scale populations have demonstrated factors that affect renal and patient survival in iMN. Moreover, we lack comparative studies of outcomes in older patients. Therefore, in this study, we investigated predictors of renal and patient outcome in iMN by analyzing Korean multicenter cohort data, with a particular focus on outcomes in older patients.

## Materials and methods

### Study population

The Korean GlomeruloNEphritis sTudy (KoGNET) included data on 21,697 patients that underwent renal biopsies from 1979 to 2018 at 18 centers in Korea. Of those patients, we retrieved data on 2,027 (9.3%) patients diagnosed with MN. Next, we excluded patients under 15 years, those with grafts or repeated biopsies, and those with MN secondary to an autoimmune disease, infectious disease, or malignancy. Our final cohort included 1,776 patients. All data were completely anonymized prior to accessing the database. This study was approved by the Ethics Committee of Kyungpook National University Hospital (IRB No. 2017-08-020).

### Data collection and outcome measurement

Demographic data were collected at the time of biopsy and at follow up. Baseline information at the time of biopsy included age, sex, body mass index, systolic and diastolic blood pressures, and comorbid conditions. Comorbid conditions included a history of hypertension, diabetes, coronary heart disease, and cerebrovascular disease. We also collected laboratory data, which included measurements of hemoglobin, total protein, albumin, blood urea nitrogen, serum creatinine, total cholesterol, low-density lipoprotein cholesterol, uric acid, immunoglobulin G, immunoglobulin A, immunoglobulin M, complement fragments C3 and C4, C-reactive protein, fasting glucose, hepatitis B surface antigen, anti-hepatitis C antibody, and the spot urine protein-to-creatinine ratio (PCR). The eGFR was calculated with the Chronic Kidney Disease Epidemiology Collaboration equation. Data were also collected at 6 months, 12 months, and at last follow up on serum creatinine and spot urine PCR. Moreover, we collected data on pathological findings from light microscopy, immunofluorescence microscopy, and electron microscopy. The iMN pathological stage was determined based on Ehrenreich and Churg criteria [24].

Clinical outcomes were proteinuria remission, renal survival, and patient survival. Complete remission was defined as a reduction in proteinuria to ≤300 mg/day. Partial remission was defined as a reduction in proteinuria to between 300 and 3,500 mg/day and a reduction greater than 50% of the baseline level. ESRD was defined by the need for renal replacement therapy, including dialysis or kidney transplantation. Maintenance renal replacement therapy and deaths were recorded between the time of biopsy and the last follow up.

### Statistical analysis

Continuous and categorical variables were compared between groups with the Student’s t-test and the Chi-square test, respectively. We performed Cox proportional hazard regression analyses to identify risk factors that affected renal and patient survival. Cumulative probabilities of complete or partial remission and survival were estimated with the Kaplan-Meier method. Both Cox proportional hazard regression and Kaplan-Meier methods tend to be biased on competing risks, because patients at risk of ESRD are evaluated for outcomes without taking into account the contributions of competing events (e.g., death). Therefore, we also performed a competing risk analysis with the Fine-Gray model.

All analyses were performed with SPSS version 19.0 (SPSS, Chicago, IL, USA) and R (R Foundation for Statistical Computing, Vienna, Austria; www.r-project.org). P-values <0.05 were considered statistically significant.

## Results

### Baseline characteristics of patients

The baseline characteristics of the 1,776 patients with iMN at the time of renal biopsy are presented in Table 1. The mean (± standard deviation) age of patients was 53.0 ± 14.7 years, and 1075 (60.5%) were male. The most common comorbid diseases were hypertension (n = 755, 46.0%) and diabetes (n = 266, 16.2%). The mean serum albumin and total cholesterol levels were 2.7 ± 0.8 g/dL and 276.4 ± 102.8 mg/dL, respectively. The mean eGFR was 86.3 ± 27.5 mL/min/1.73 m^2^. The mean urine PCR at the time of renal biopsy was 5.56 ± 4.52 g/g, and 871 (49.0%) patients had nephrotic-range proteinuria (≥3.5 g/g).

**Table 1:** Baseline characteristics of patients with idiopathic membranous nephropathy at the time of renal biopsy.

Characteristic	Total	Age <65 y	Age ≥65 y	p
	(n = 1776)	(n = 1348)	(n = 428)	
Sex (male)	1075 (60.5%)	829 (61.5%)	246 (57.5%)	0.154
Age (years)	53.0 ± 14.7	47.2 ± 11.7	71.2 ± 5.2	<0.001*
Body mass index (kg/m^2^)	24.8 ± 3.7	24.9 ± 3.8	24.5 ± 3.3	0.114
Systolic BP (mmHg)	126.8 ± 17.8	126.1 ± 17.4	129.1 ± 19.1	0.003*
Diastolic BP (mmHg)	78.6 ± 12.6	79.4 ± 12.9	76.1 ± 11.6	<0.001*
Comorbid conditions				
Hypertension	755 (42.5%)	489 (36.3%)	266 (62.1%)	<0.001*
Diabetes	266 (15.0%)	153 (11.4%)	113 (26.4%)	<0.001*
Coronary Heart Disease	55 (3.1%)	33 (2.4%)	22 (5.1%)	0.043*
Cerebrovascular Disease	83 (4.7%)	41 (3.0%)	42 (9.8%)	<0.001*
Hemoglobin (g/dL)	13.1 ± 2.0	13.4 ± 1.9	12.2 ± 1.9	<0.001*
Total protein (g/dL)	5.3 ± 1.0	5.3 ± 1.1	5.20 ± 1.0	0.221
Albumin (g/dL)	2.7 ± 0.8	2.8 ± 0.8	2.6 ± 0.7	<0.001*
Blood urea nitrogen (mg/dL)	17.1 ± 11.4	15.7 ± 10.4	20.8 ± 13.1	<0.001*
Serum creatinine (mg/dL)	1.0 ± 0.8	1.0 ± 0.8	1.2 ± 0.9	<0.001*
eGFR (ml/min/1.73 m^2^)	86.3 ± 27.5	92.1 ± 26.0	68.0 ± 23.9	<0.001*
Total Cholesterol (mg/dL)	276.4 ± 102.8	283.7 ± 106.1	254.5 ± 88.9	<0.001*
LDL (mg/dL)	172.3 ± 84.9	177.9 ± 88.1	158.6 ± 74.9	0.006*
Uric acid	6.1 ± 1.8	6.1 ± 1.8	6.3 ± 1.9	0.028*
C3 (mg/dL)	116.2 ± 28.9	115.1 ± 29.1	119.6 ± 28.3	0.007*
C4 (mg/dL)	31.7 ± 12.6	31.5 ± 13.1	32.3 ± 10.8	0.306
CRP (mg/dL)	1.1 ± 4.1	1.0 ± 4.0	1.5 ± 4.1	0.063
Glucose (mg/dL)	111.0 ± 34.1	109.5 ± 34.2	114.7 ± 33.7	0.013*
Urine P-CR (g/g)	5.56 ± 4.52	5.14 ± 4.32	6.79 ± 4.89	<0.001*
≥3.5 g/g	871 (49.0%)	612 (45.4%)	259 (60.5%)	
<3.5 g/g	576 (32.4%)	468 (34.7%)	108 (25.2%)	
Histology stage				0.262
I	189 (27.8%)	136 (27.5%)	53 (28.3%)	
II	209 (30.7%)	151 (30.6%)	58 (31.0%)	
III	96 (14.1%)	71 (14.4%)	25 (13.4%)	
IV	10 (1.5%)	6 (1.2%)	4 (2.1%)	
V	2 (0.3%)	0 (0.0%)	2 (1.1%)	
Mixed stage	175 (25.7%)	130 (26.3%)	45 (24.1%)	

Values are expressed as the mean ± SD, or number (%).

*p <0.05 for age <65 y vs. age ≥65 y; BP, blood pressure; CRP, C-reactive protein; eGFR, estimated glomerular filtration rate; LDL, low-density lipoproteins; PCR, protein-creatinine ratio.

When we grouped patients according to ages over 65 y (older group) and ages under 65 y (younger group), we observed that systolic blood pressure was significantly higher in the older group than in the younger group (129.1 ± 19.1 vs. 126.1 ± 17.4 mmHg), but diastolic blood pressure was significantly lower in the older group than in the younger group (76.1 ± 11.6 vs. 79.4 ± 12.9 mmHg). The older group had a higher prevalence of comorbidities, including hypertension, diabetes, coronary heart disease, and cerebrovascular disease, compared to the younger group. The hemoglobin, serum albumin, and total cholesterol levels were significantly lower in the older group compared to the younger group. The eGFR was also significantly lower in the older group than in the younger group (68.0 ± 23.9 vs. 92.1 ± 26.0 mL/min/1.73 m^2^). More patients in the older group (60.5%) had nephrotic-range proteinuria than in the younger group (45.4%). There was no significant difference in the distribution of histology stages between the older and younger groups.

### Remission of proteinuria

**Fig 1:**
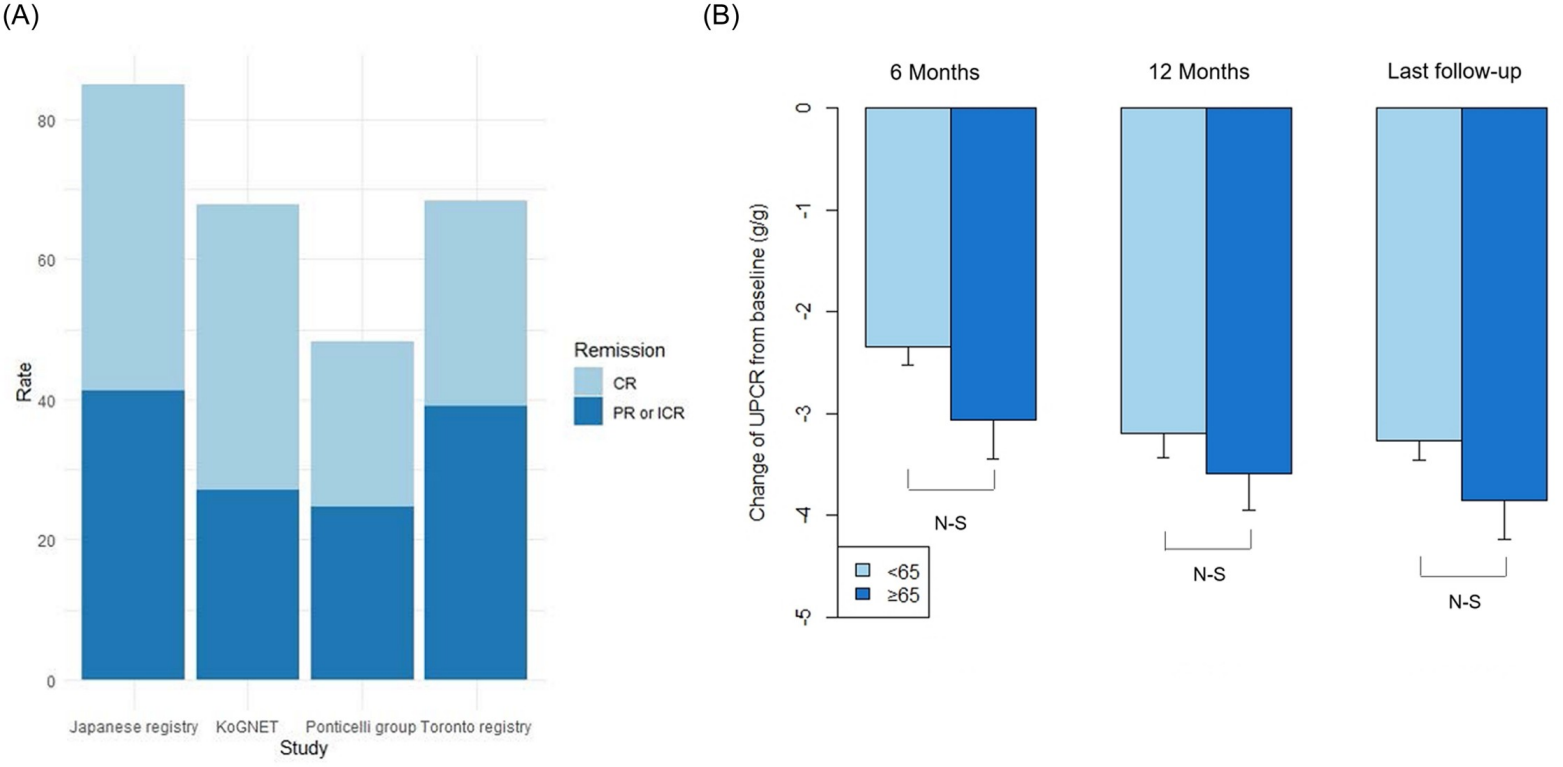
Complete or partial remission rate and changes in proteinuria among patients with idiopathic membranous nephropathy. (A) The complete or partial remission rate at last follow-up was 68.0% in the KoGNET database, which was similar to the rate in the Toronto registry (68.4%), lower than the rate in the Japanese registry (85.0%), and higher than the rate in the Ponticelli group (48.2%). (B) Changes in proteinuria from the time of biopsy were not significantly different between the older and younger groups at 6 months, 12 months, and at last follow up. CR, complete remission; ICR, incomplete remission; PR, partial remission; UP-CR, spot urine protein-to-creatinine ratio.

The median follow-up duration was 88.0 months (interquartile range [IQR]: 38.0–146.0). Complete or partial remission rates were 48.5%, 63.8%, and 68.0% at 6 and 12 months after biopsy, and at the last follow up, respectively (Table 2). Complete remission rates at 6 and 12 months after biopsy and at the last follow up were 15.7%, 28.0%, and 40.8% (Fig 1A), respectively. Partial remission rates at those time points were 32.8%, 35.7%, and 27.1%, respectively. Complete or partial remission rates at 12 months were significantly lower in the older group (53.5%) compared to the younger group (67.5%; p = 0.002). However, these rates were not significantly different between the two groups at 6 months (47.8% vs. 48.8%, p = 0.872) or at last follow up (62.9% vs. 70.1%, p = 0.066). Changes in proteinuria from the time of biopsy were not significantly different between the older and younger groups at 6 months (−3.1 ± 5.6 vs. −2.3 ± 4.1 g/g, p = 0.062), 12 months (−3.6 ± 4.5 vs. −3.2 ± 4.8 g/g, p = 0.382), or at the last follow up (−3.9 ± 5.7 vs. −3.3 ± 4.5 g/g, p = 0.139; Fig 1B).

**Table 2:** Proteinuria remission at 6 and 12 months and at last follow-up in patients with idiopathic membranous nephropathy.

Time point	Status	Total	Age <65 y	Age ≥65 y	p
6 Months	Remission	345 (48.5%)	247 (48.8%)	98 (47.8%)	0.872
	CR	112 (15.7%)	80 (15.8%)	32 (15.6%)	
	PR	233 (32.8%)	167 (33.0%)	66 (32.2%)	
	No remission	366 (51.5%)	259 (51.2%)	107 (52.2%)	
12 Months	Remission	380 (63.8%)	295 (67.5%)	85(53.5%)	0.002*
	CR	167 (28.0%)	131 (30.0%)	36 (22.6%)	
	PR	213 (35.7%)	164 (37.5%)	49 (30.8%)	
	No remission	216 (36.2%)	142 (32.5%)	74 (46.5%)	
Last follow-up	Remission	516 (68.0%)	377 (70.1%)	139 (62.9%)	0.066
	CR	310 (40.8%)	233 (43.3%)	77 (34.8%)	
	PR	206 (27.1%)	144 (26.8%)	62 (28.1%)	
	No remission	243 (32.0%)	161 (29.9%)	82 (37.1%)	

*p value <0.05 for age <65 y vs. age ≥65 y; CR, complete remission; PR, partial remission.

### Renal outcome

**Fig 2:**
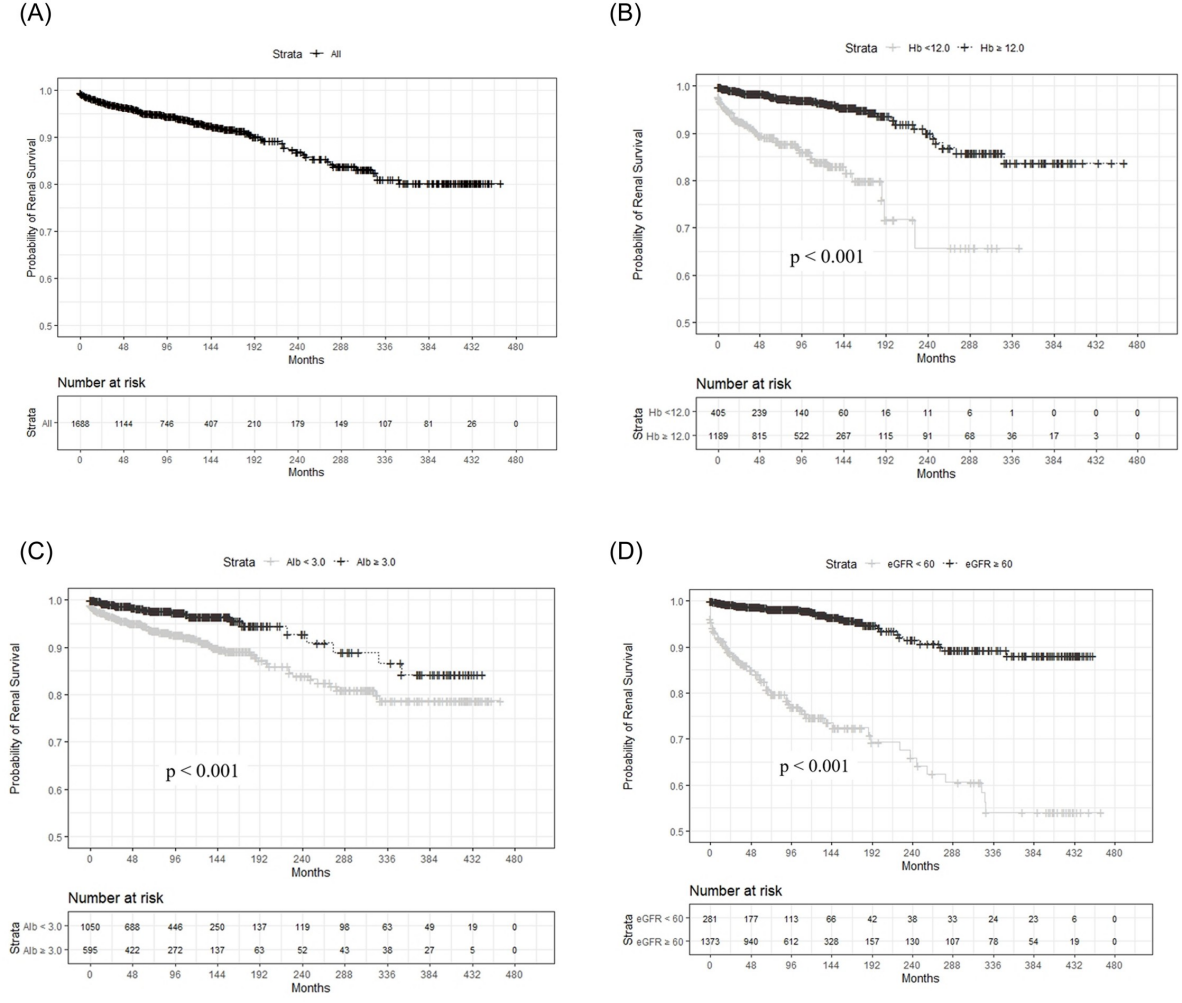
Renal survival in idiopathic membranous nephropathy, analyzed with the Kaplan-Meier method. (A) The 1-year, 5-year, and 10-year renal survival rates of all patients were 98.3%, 95.9%, and 93.4%, respectively. (B-D) Patients with higher hemoglobin (≥12.0 g/dL) (B), albumin (≥3.0 g/dL) (C), and eGFR (≥60 ml/min/1.73 m^2^) (D) had significantly better renal survival compared to patients with lower values.

During the follow-up period, 112 patients (6.3%) progressed to ESRD. The median renal survival time was 82.5 months (IQR: 35.8–109.3). The 1-year, 5-year, and 10-year renal survival rates were 98.3%, 95.9%, and 93.4%, respectively (Fig 2A).

Cox proportional hazard regression analyses were conducted to evaluate renal survival. The univariate analysis showed that older age (hazard ratio [HR]: 1.02, 95% confidence interval [CI]: 1.01–1.04, p <0.001), the presence of hypertension (HR: 2.21, 95%CI: 1.46–3.36, p <0.001), and diabetes (HR: 1.97, 95%CI: 1.22–3.18, p = 0.006) at biopsy were significant risk factors for ESRD. In contrast, high levels of hemoglobin (HR: 0.70, 95%CI: 0.63–0.76, p <0.001), albumin (HR: 0.59, 95%CI: 0.45–0.77, p <0.001), and eGFR (HR: 0.94, 95%CI: 0.93–0.95, p <0.001) were good prognostic factors for renal outcomes.

**Fig 3:**
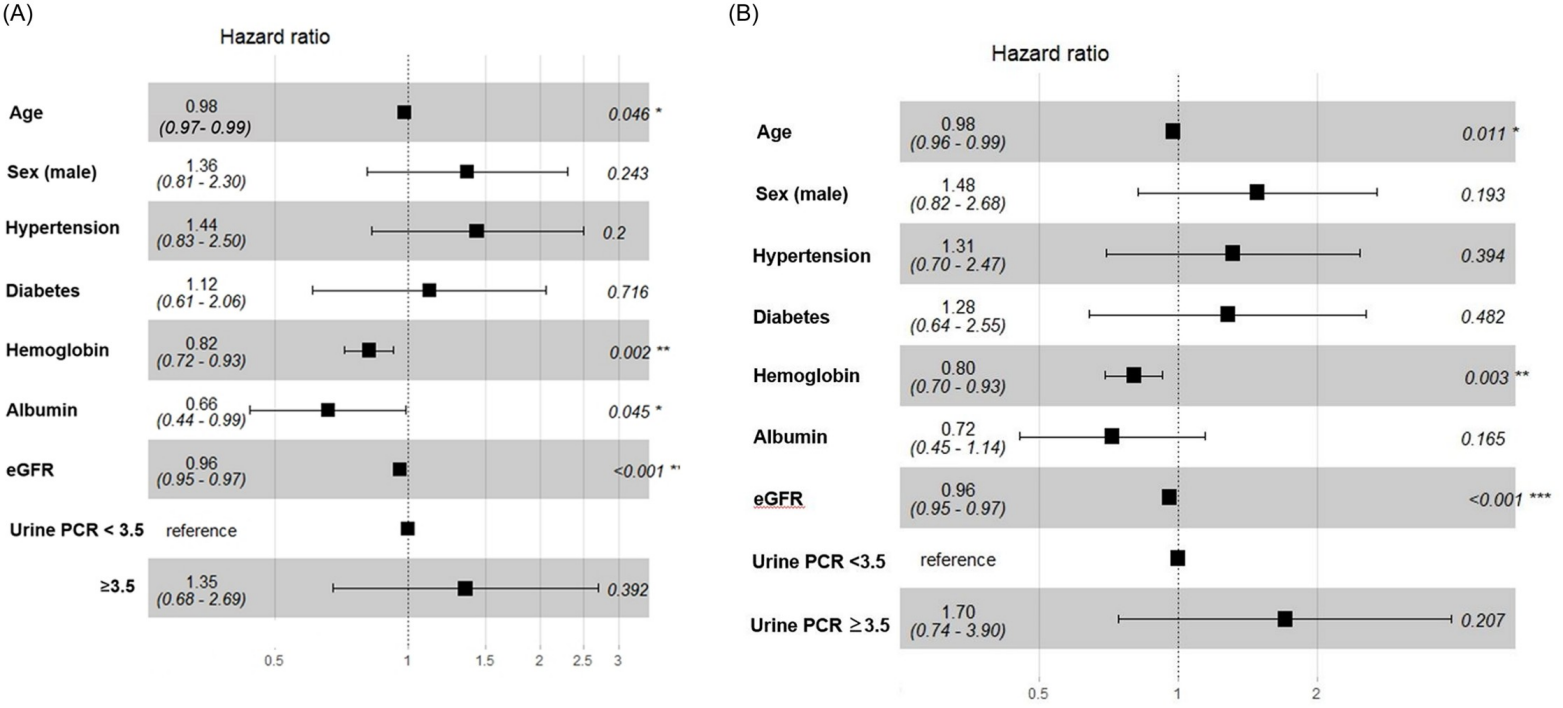
Predictors of renal survival in idiopathic membranous nephropathy. (A) Multivariate Cox regression analysis identified older age, high hemoglobin, high albumin, and high eGFR at biopsy as good prognostic factors for renal survival. (B) Similar results, except for albumin, were obtained after accounting for competing risk events, ESRD and death.

In the multivariate regression analysis, we found that high levels of hemoglobin (HR: 0.82, 95%CI: 0.72–0.93, p = 0.002), albumin (HR: 0.66, 95%CI: 0.44–0.99, p = 0.045), and eGFR (HR: 0.96, 95%CI: 0.95–0.97, p <0.001) at biopsy remained good predictors of renal outcomes. Interestingly, older age (HR: 0.98, 95%CI: 0.97–0.99, p = 0.046) was also a good prognostic factor for renal survival, after adjusting for multiple variables (Fig 3A). Next, we applied the Fine-Gray model to take into consideration the two main competing risk events, ESRD and death. We found that high levels of hemoglobin (HR: 0.80, 95%CI: 0.70–0.93, p = 0.003), eGFR (HR: 0.96, 95%CI: 0.95–0.97, p <0.001]), and age (HR: 0.98, 95%CI: 0.96–0.99, p = 0.011) at biopsy remained good prognostic factors for renal outcomes (Fig 3B).

Kaplan-Meier curves revealed that patients with high levels of hemoglobin (≥12.0 g/dL), albumin (≥3.0 g/dL), and eGFR (≥60 mL/min/1.73 m^2^) had significantly better renal survival compared to patients with lower values (Fig 2B–2D).

### Patient survival

**Fig 4:**
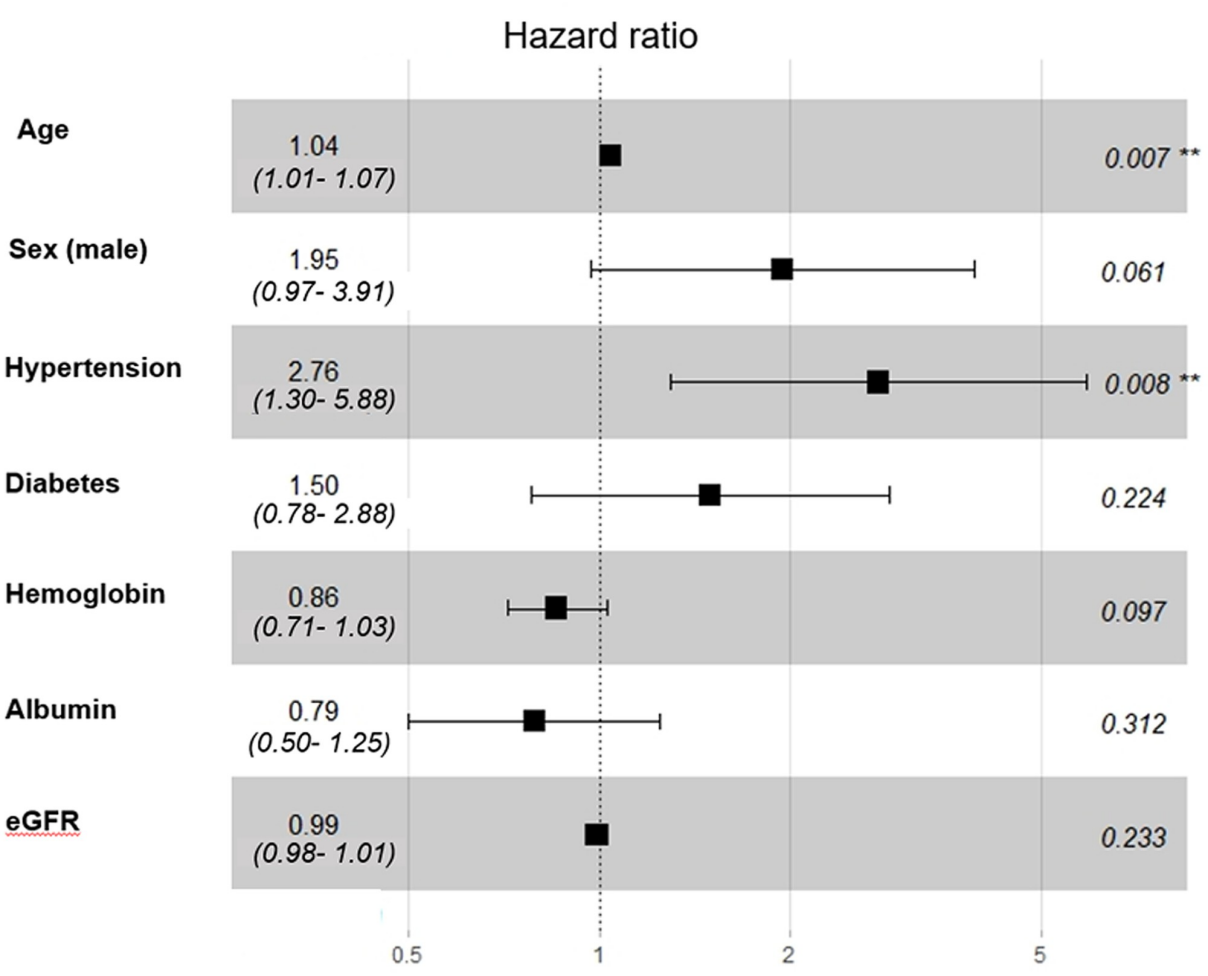
Predictors of patient survival from idiopathic membranous nephropathy. Multivariate Cox proportional regression results show that older age and hypertension at biopsy were significant risk factors for patient survival.

The Cox proportional hazard model showed that older age (HR: 1.07, 95%CI: 1.05–1.10, p <0.001), hypertension (HR: 4.61, 95%CI: 2.25–9.44, p <0.001), and diabetes (HR: 3.01, 95%CI: 1.60–5.64, p = 0.001) at biopsy were poor predictors of patient survival. In contrast, the univariate analysis indicated that high levels of hemoglobin (HR: 0.76, 95%CI: 0.66–0.88, p <0.001) and eGFR (HR: 0.97, 95%CI: 0.96–0.98, p <0.001) were good predictors of patient survival. After adjusting for multiple variables in a multivariate Cox proportional regression analysis, we found that older age (HR: 1.04, 95%CI: 1.01–1.07, p = 0.007) and hypertension at biopsy (HR: 2.76, 95%CI: 1.30–5.88, p = 0.008) were significant risk factors for patient survival (Fig 4).

## Discussion

In this study, we accessed data from a nation-wide multicenter database to investigate factors that affected renal and patient outcomes in iMN, with a focus on older patients. Several previous studies have included 200–300 patients with MN [10, 17, 25], but few large-scale studies with more than 1,000 patients have been published. In our cohort, iMN was found in 9.3% of biopsies collected over a span of 40 years. This rate was similar to rates reported in other countries [26, 27]. However, the mean age of patients in our Korean KoGNET cohort was 53 years, which was older than the cohorts studied in other countries, including Japan (mean age 50.5 y) [16], China (mean age 43.9 y) [25], Italy (mean age 44.9 y), and Canada (mean age 42.5 y) [28]. Moreover, the proportion of our cohort with hypertension (46.0%) was higher than those reported in Japanese (24.7%) and Chinese (29.8%) cohorts, but similar to that reported in a cohort from the United States (47.4%) [25, 29, 30]. In addition, we found significant differences between older and younger patients in many clinical parameters, including serum albumin, eGFR, and nephrotic-range proteinuria, as well as comorbidities, such as hypertension and diabetes. In contrast, among 323 patients diagnosed with iMN in the Toronto Glomerulonephritis Registry data, no differences were observed between older and younger groups in serum albumin or in the percentage of patients with nephrotic-range proteinuria [31]. More studies are needed to determine whether the discrepancies between these studies might arise from geographic or other differences between the two cohorts.

Previous studies reported complete or partial remission rates of 55%–68% in patients with iMN [14, 18]. Consistent with those data, we found complete or partial remission rates of 63.8% at 12 months and 68.0% at last follow up (median 88 months; Fig 1). Similarly, the Toronto registry cohort showed a 68.4% complete or partial remission rate over a median follow up of 60 months [18]. However, other cohorts showed higher or lower remission rates. The Japanese registry showed an 85.0% complete or incomplete remission rate over a mean 83.3-month follow-up period) [16]. In contrast, the Ponticelli group showed a 48.2% complete or partial remission rate in treated and untreated groups over a 10-year follow-up period [32]. In our cohort, remission rate at 12 months was significantly lower in the older group (53.5%) compared to the younger group (67.5%), but there was no significant difference between groups at 6 months or at the last follow up. In contrast, the Toronto registry study showed similar remission rates in the older (20%) and younger (16%) groups [31]. Our finding of a lower remission rate in the older group than in the younger group at 12 months might be related to the higher proteinuria levels at the time of biopsy in older compared to younger patients. However, changes in proteinuria from the time of biopsy to 6 months, 12 months, and the last follow up were not significantly different between the older and younger groups. Remission rates and reductions in proteinuria might vary with registry, age, or use of immunosuppressive agents. Further studies are warranted to address this issue.

Renal survival in this KoGNET iMN cohort was 93.4% in the 10th year, which was similar to the rate observed in a Chinese cohort (93.5%) [17], somewhat higher than the rate observed in a Japanese cohort (90.3%), and higher than the rate observed in a Caucasian cohort (80%) [16, 33]. A number of previous studies confirmed the factors that determined long-term renal outcomes in iMN and identified candidate predictors, including heavy proteinuria, the duration of proteinuria, hypertension, old age, male gender, renal impairment, and greater tubulointerstitial disease [16, 33–35]. Because iMN occurs frequently in older patients, many investigators are interested in the relationship between renal outcomes and age. Although some studies have shown that older patients with iMN had a poor prognosis [8, 16, 17], a recent study that analyzed Japanese data showed that younger and older patients with iMN had similar renal outcomes [15]. Generally, at the time of biopsy, the initial eGFR is lower in older patients than in younger patients; consequently, older patients might reach renal failure faster than younger patients. However, Zent *et al*. showed that, among patients with iMN in the Toronto registry, the rates of creatinine clearance decline were not different between the older and younger age groups. Therefore, they suggested that the renal prognosis in older patients might be influenced by the underlying condition of patients at the time they presented with the disease, rather than disease severity [31]. Interestingly, in our cohort, older patients had better renal outcomes than younger patients, even though older patients had more comorbidities, such as hypertension and diabetes, than younger patients. Moreover, our cohort had a higher mean age than cohorts of other registries. Thus, our results also suggested that poor renal outcomes did not necessarily depend solely on age, particularly after adjusting for multiple variables. This finding was consistent with those reported in the Toronto registry study. Additionally, in this study, we performed a competing risk analysis to account for the likelihood that the time to ESRD would be shorter in older patients than in younger patients, because older individuals had a shorter life expectancy and a higher risk of comorbidity-related death than younger patients. Nevertheless, we found that the association between older age and favorable renal outcomes remained significant after accounting for competing risks. Our multivariate analysis showed that sex, hypertension, diabetes, and nephrotic-range proteinuria were not significant predictors for progression to ESRD. In contrast, high hemoglobin, high albumin, and high eGFR were good prognostic factors. These findings reflected the notion that renal survival is likely to be higher among patients in good general health.

Older age was previously related to inferior patient survival among patients that required a renal biopsy [36]. In our cohort, older age and the presence of hypertension were significant risk factors for patient survival. However, predictors of patient mortality might depend on race and registry. In our study, no data on the cause of death were available. Therefore, further studies are needed to confirm these results.

This study had several limitations. First, this was a retrospective study; thus, we might have lacked access to some relevant data. Second, we collected KoGNET data over a relatively long time, and treatment protocols might have changed over the years. Therefore, outcomes might be difficult to interpret. Third, we lacked data on medications, such as immunosuppressive agents, which could have affected the outcomes. Nevertheless, our cohort included a large number of patients with iMN, and outcomes were recorded over a long period of time. Thus, our findings could be expected to provide a basis for predicting prognoses and establishing optimal management in patients with iMN.

In conclusion, this study showed that, among patients with iMN, older patients had more favorable renal outcomes, but worse patient survival than younger patients. We found that high hemoglobin, high serum albumin, and good renal function at biopsy were good predictors of renal survival in patients with iMN. We also found that hypertension at biopsy was a poor prognostic factor for patient survival in iMN. More longitudinal, large-scale, comparative studies are needed to provide more prognostic indicators for patients with iMN.
